# Coming out of the Shell: Building the Molecular Infrastructure for Research on Parasite-Harbouring Snails

**DOI:** 10.1371/journal.pntd.0002284

**Published:** 2013-09-12

**Authors:** Cinzia Cantacessi, Sattrachai Prasopdee, Javier Sotillo, Jason Mulvenna, Smarn Tesana, Alex Loukas

**Affiliations:** 1 Centre for Biodiscovery and Molecular Development of Therapeutics, James Cook University, Smithfield, Queensland, Australia; 2 Department of Parasitology, Khon Kaen University, Khon Kaen, Thailand; George Washington University School of Medicine and Health Sciences, United States of America

In Thailand and Laos alone, approximately 10 million people are infected with the liver fluke *Opisthorchis viverrini* [1]. Chronic infection with this parasite is considered the leading cause of cholangiocarcinoma (CCA, or bile-duct cancer) in large areas of Southeast Asia [2]. In these regions, CCA caused by *O. viverrini* is typically diagnosed 30–40 years after infection, with death occurring within 3–6 months post diagnosis [3]. *O. viverrini* is characterised by a three-host life cycle, with prosobranch snails of the genus *Bithynia* and cyprinid fishes acting as first and second intermediate hosts, respectively, while piscivorous mammals, including dogs, cats, and humans, act as definitive hosts [2]. Over the last two decades, much attention has been paid to studies on the epidemiology, developmental biology, and diagnosis of *O. viverrini* [4], while recent biotechnological advances are contributing large-scale explorations of the fundamental molecular biology of this liver fluke, with a view toward identifying key molecules essential for its development, reproduction, and survival, as well as dissecting the molecular pathways leading to the development of CCA [5]–[8]. These advances provide a solid foundation for the development of novel strategies to fight this devastating disease. However, long-term control of *O. viverrini*–induced cancer strictly relies on the development of integrated approaches, targeting the parasite as well as its intermediate hosts.

Recently, an article in *PLOS Neglected Tropical Diseases* by Adema and colleagues [9] served to highlight the substantial gap in knowledge of aspects of the fundamental molecular biology of molluscs harbouring parasites, as well as the extraordinary opportunities that modern research toolkits, including microarray platforms, RNA interference, and high-throughput sequencing, offer for investigations of snail-parasite interactions [9]. Indeed, despite the massive expansion in the demand for and access to low-cost, high-throughput sequencing, large-scale genomic analyses of snails are limited to the draft genome sequence of the pulmonate snail intermediate host of *Schistosoma* blood flukes (*Biomphalaria glabrata* Genome Initiative at http://biology.unm.edu/biomphalaria-genome/index.html). Until now, there has been no genomic or transcriptomic information available for the prosobranch snail intermediate hosts of carcinogenic liver flukes, such as *O. viverrini* and *Clonorchis sinensis*. In order to provide the research community with a solid resource for molecular studies of these organisms, we generated the first reference transcriptome of *Bithynia siamensis goniomphalos* (Gastropoda, Bithyniidae), the intermediate host of *O. viverrini* in areas of northeast Thailand, Lao PDR, Cambodia, and South Vietnam, where the incidence of CCA is highest [cf. 10] (cf. Figure 1). A cDNA library from adult snails [11] was constructed, sequenced using RNA-seq (Illumina), and annotated using an established bioinformatic workflow [12]. Briefly, snails were collected from a natural body of water in Muang District, Khon Kaen Province, northeast Thailand (Figure 1); the taxonomic identity of the specimens was confirmed based on characteristic morphological features of the shells [13]; RNA was extracted from whole adult, parasite-free *B. siamensis goniomphalos* (n = 5), reverse-transcribed to cDNA, adaptor-ligated, and paired-end sequenced on a Genome Analyzer II (Illumina). Almost 50 million high-quality (Phred score >28) reads were generated; the assembly, produced using the Trinity software [14], yielded 167,029 contigs >200 bp in length, with a GC content of 44.4% (Table 1). Using sequence homology–based searches [12], approximately 40% of the assembled contigs could be annotated (cf. Table 1). A total of 32,026 contigs could be annotated with Gene Ontology terms (*via* Blast2GO; [15]), according to the categories “biological process,” “cellular component,” and “molecular function.” Approximately 77,000 non-overlapping protein sequences (of which ∼15,000 were full-length) could be inferred from the transcriptome of *B. siamensis goniomphalos via* BLASTx alignments with protein sequences available in public databases. Of the *B. siamensis goniomphalos* transcripts encoding proteins that could be mapped to orthologues in the Clusters of Orthologous Groups (COGs) database [16] (Table 1), the largest set was assigned to “general function prediction only” (16%), followed by “translation, ribosomal structure and biogenesis” (10%), “replication, recombination and repair” (7%), and “transcription” (7%) (Table 1). Approximately 54,000 protein-coding transcripts had orthologues in one of the 29 known biological pathways in the KEGG database (Table 1), including “regulation of actin cytoskeleton” (4%), “focal adhesion” (4%), and “spliceosome” (3.9%) (Table 1). Among the major protein classes included in the *B. siamensis goniomphalos* transcriptome were peptidases (n = 1,944; 4%), kinases (n = 4,757; 9%), phosphatases (n = 2,151; 4%), GTPases (n = 2,533; 5%), receptors (n = 6,243; 11%), transcription factors (n = 1,478; 3%), and channels and transporters (n = 1,287; 2%). Annotation information linked to each transcript characterised in the present study, including top BLASTx hit, GO classification, KEGG pathway mapping, and COG orthologues, is available from Table S1. In the absence of a reference genome for *Bithynia* spp., the annotation of the sequence data analysed herein was based on comparison with data available in public databases. The relatively small proportion of *B. siamensis goniomphalos* annotated protein sequences (∼60%) is likely to reflect the paucity of genomic sequence information available for prosobranch molluscan species in these databases [17]; supported by the availability of this dataset, as well as of the draft genome sequence of *B. glabrata*, future sequencing efforts will provide the depth of coverage required for the determination of the genome of *Bithynia* spp., which, in turn, will pave the way for comparative genomic studies of prosobranch and pulmonate snails harbouring parasite infections. This knowledge will be pivotal to improve our understanding of the biology of snail-borne parasites, and their “choice” of distinct snail species as their intermediate hosts.

**Figure 1:**
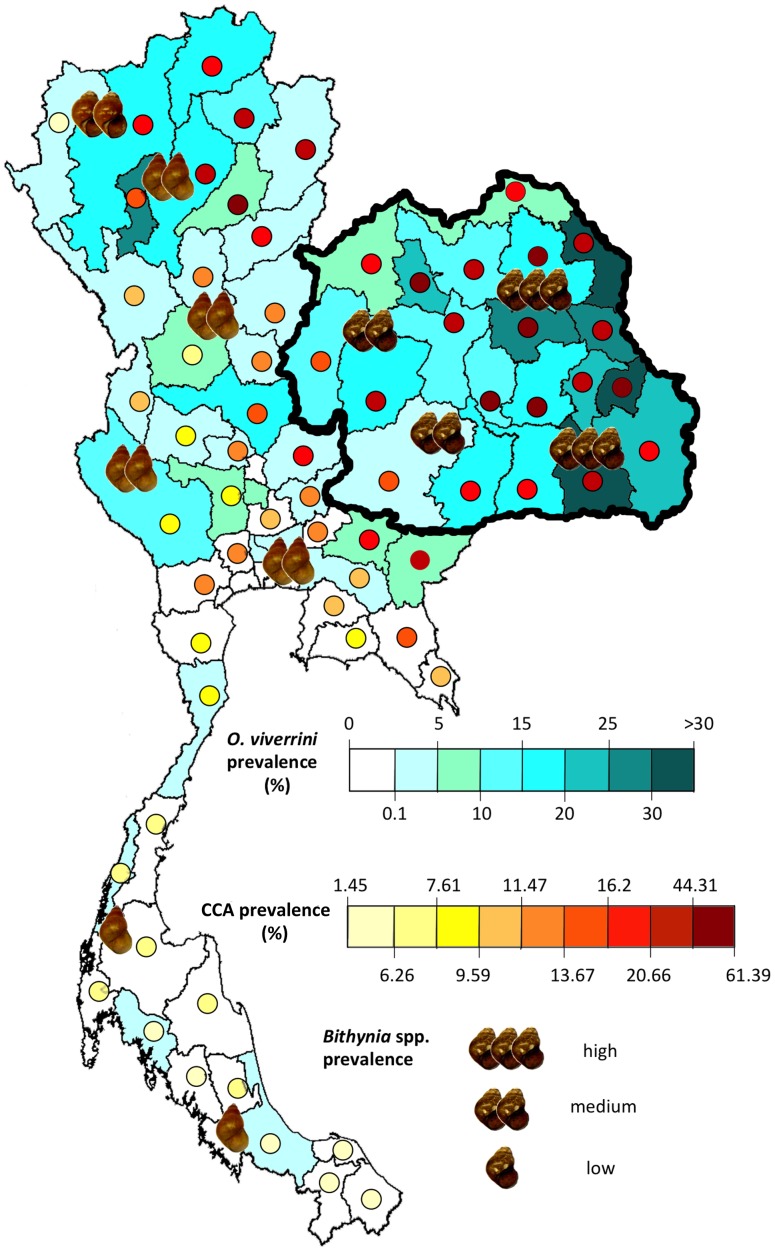
Distribution and prevalence of *Opisthorchis viverrini*, cholangiocarcinoma, and *Bithynia* spp. snails in Thailand. At present, three species of *Bithynia* act as intermediate hosts for *O. viverrini*, and their distribution differs all over Thailand. *B. siamensis goniomphalos* is located in northeast Thailand (thick border), where the prevalence of *O. viverrini*–associated cholangiocarcinoma (CCA) is highest; *B. funiculata* specifically distributes in north Thailand; and *B. siamensis siamensis* is mainly found in central and north Thailand, and rarely in the south [2], [18]–[20].

**Table 1:** Summary of the RNA-seq data for *Bythinia siamensis goniomphalos* prior to and following assembly, and bioinformatics annotation and analyses.

*Raw reads (paired-end)*	49,952,248
Total contigs (average length ± SD)	167,029 (710±677)
GC content (%)	44.4
Number of BLASTn hits* (%)	47,220 (46)
Number of BLASTx hits* (%)	77,714 (77)
Containing an open reading frame (%)	76,963
Returning a COG result (%)	54,513 (71)
KEGG pathway result (%)	54,291 (70.5)
Gene Ontology results (%)	32,026 (41.6)

* e-value cut-off: 1e-05.

Both raw and assembled sequence data generated in the present study are freely available from the Sequence Read Archive (SRA) and the Transcriptome Shotgun Assembly Sequence Database at NCBI (http://www.ncbi.nlm.nih.gov) under accession numbers SRR768418 and GAGS0000000, respectively. To our knowledge, this sequence data represents the first large-scale transcriptomic resource for a prosobranch mollusc intermediate host of a human platyhelminth and represents a major contribution to future fundamental explorations of the developmental biology of *O. viverrini* in *Bithynia* spp. snails, as well as the molecular interactions occurring at the snail-parasite interface. We suggest that the extensive sequence data generated in this study will be of great value in future studies aimed at exploring the changes in gene transcription occurring in *Bithynia* spp. upon infection with *O. viverrini* eggs and at different time points following infection. Besides yielding a general picture of the modified biology of trematode-infected snails, this data will set a basis for the identification of key genes, gene products, biological pathways, and/or bacterial and viral symbionts [cf. 9] involved in the cascade of molecular events leading to the development of the parasite through the stages of miracidium, sporocyst, redia, and cercaria. In turn, this advance will ultimately result in the development of novel targeted strategies to control snail-borne NTDs.

New molecular technologies have a tremendous potential to aid our efforts in controlling snail-borne diseases. However, despite the unanimous acknowledgment that an increased understanding of the fundamental molecular biology of parasite-harbouring snails will provide us with a range of new tools to aid current efforts aimed at controlling snail-borne infections, the progressive but steady decline of malacology expertise and funding for snail vector–related research [cf. 9] poses a serious obstacle to the application of such technologies to snail-oriented NTD control. With this first step toward the establishment of a reference database for genetic research of prosobranch snail vectors of parasitic helminths, we hope to stimulate integrated, interdisciplinary research across malacology, helminthology, genomics, and bioinformatics, in the bid to fight snail-borne infections.

## Supporting Information

Table S1 **The annotated transcriptome of** ***Bithynia siamensis goniomphalos*** **.** Summary of annotation data linked to individual assembled transcripts. Abbreviations: reads per kilobase per million reads (RPKM); non-redundant protein database (Nr); non-redundant nucleotide database (Nt); SwissProt database (SwissProt); Clusters of Orthologous Groups of Proteins (COG); Kyoto Encyclopedia of Genes and Genomes (KEGG); Gene Ontology (GO).
(ZIP)
